# Novel truncating mutations in *CTNND1* cause a dominant craniofacial and cardiac syndrome

**DOI:** 10.1093/hmg/ddaa050

**Published:** 2020-03-20

**Authors:** Reham Alharatani, Athina Ververi, Ana Beleza-Meireles, Weizhen Ji, Emily Mis, Quinten T Patterson, John N Griffin, Nabina Bhujel, Caitlin A Chang, Abhijit Dixit, Monica Konstantino, Christopher Healy, Sumayyah Hannan, Natsuko Neo, Alex Cash, Dong Li, Elizabeth Bhoj, Elaine H Zackai, Ruth Cleaver, Diana Baralle, Meriel McEntagart, Ruth Newbury-Ecob, Richard Scott, Jane A Hurst, Ping Yee Billie Au, Marie Therese Hosey, Mustafa Khokha, Denise K Marciano, Saquib A Lakhani, Karen J Liu

**Affiliations:** 1 Centre for Craniofacial and Regenerative Biology, Faculty of Dentistry, Oral and Craniofacial Sciences, King's College London, London SE1 9RT, UK; 2 Paediatric Dentistry, Centre of Oral, Clinical and Translational Science, Faculty of Dentistry, Oral and Craniofacial Sciences, King's College London, London SE5 9RS, UK; 3 Department of Clinical Genetics, Great Ormond Street Hospital Trust, London WC1N 3JH, UK; 4 Department of Clinical Genetics, Guy’s and St. Thomas’ NHS Foundation Trust, London SE1 9RT, UK; 5 Pediatric Genomics Discovery Program, Department of Pediatrics, Yale University School of Medicine, New Haven, CT 06520, USA; 6 Departments of Internal Medicine and Cell Biology, University of Texas Southwestern Medical Center, Dallas, TX 75390-8856, USA; 7 Pediatric Genomics Discovery Program, Departments of Genetics and Pediatrics, Yale University School of Medicine, New Haven, CT 06520, USA; 8 South Thames Cleft Service, Guy’s and St. Thomas’ NHS Foundation Trust, London SE1 7EH, UK; 9 Department of Medical Genetics, Cumming School of Medicine, Alberta Children’s Hospital Research Institute, University of Calgary, AB, Canada; 10 Nottingham University Hospitals NHS Trust, Nottingham NG5 1PB, UK; 11 Tokyo Medical and Dental University, Tokyo, Japan; 12 Center for Applied Genomics, Children’s Hospital of Philadelphia, Philadelphia, PA 19104, USA; 13 Department of Pediatrics, Division of Human Genetics, Children's Hospital of Philadelphia, Philadelphia, PA 19104, USA; 14 Peninsula Clinical Genetics Service, Royal Devon and Exeter NHS Foundation Trust, Exeter EX2 5DW, UK; 15 Human Development and Health, Faculty of Medicine, University of Southampton, Southampton SO17 1BJ, UK; 16 Department of Clinical Genetics, St George's Hospital, London SW17 0RE, UK; 17 Clinical Genetics, University Hospital Bristol NHS Foundation Trust, Bristol BS2 8EG, UK

## Abstract

*CTNND1* encodes the p120-catenin (p120) protein, which has a wide range of functions, including the maintenance of cell–cell junctions, regulation of the epithelial-mesenchymal transition and transcriptional signalling. Due to advances in next-generation sequencing, *CTNND1* has been implicated in human diseases including cleft palate and blepharocheilodontic (BCD) syndrome albeit only recently. In this study, we identify eight novel protein-truncating variants, six *de novo,* in 13 participants from nine families presenting with craniofacial dysmorphisms including cleft palate and hypodontia, as well as congenital cardiac anomalies, limb dysmorphologies and neurodevelopmental disorders. Using conditional deletions in mice as well as CRISPR/Cas9 approaches to target *CTNND1* in *Xenopus*, we identified a subset of phenotypes that can be linked to p120-catenin in epithelial integrity and turnover, and additional phenotypes that suggest mesenchymal roles of *CTNND1.* We propose that *CTNND1* variants have a wider developmental role than previously described and that variations in this gene underlie not only cleft palate and BCD but may be expanded to a broader velocardiofacial-like syndrome.

## Introduction

Genetic variation in *CTNND1*, which encodes for the armadillo-repeat protein p120-catenin (p120), is associated with human birth defects, most notably non-syndromic cleft palate and blepharocheilodontic (BCD) syndrome, which involves eyelid, lip and tooth anomalies [MIM: 617681] (1–3). In contrast, experiments in animal models have suggested broader developmental roles for *CTNND1*. For example, conditional deletions in mice demonstrate the importance of *CTNND1* for development not only for skin and teeth but also for kidneys and other structures (4–10), and the complete deletion of *CTNND1* leads to prenatal lethality (5,9). Similarly, loss-of-function experiments in *Xenopus* implicate *CTNND1* in craniofacial development (11,12). Here, we describe a series of patients with *CTNND1* variants, all of whom present with multisystem involvement that demonstrates a broad spectrum craniofacial and cardiac syndrome.

p120-catenin is a member of the catenin superfamily of proteins studied in catenin–cadherin interactions; notably, it binds to and stabilizes E-cadherin (*CDH1*) at junctional complexes in epithelia (13–17). This binding is via the p120-catenin armadillo repeat domain, and the displacement of p120-catenin from E-cadherin is a key regulatory event at the adherens junction, which results in the endocytosis of E-cadherin and loss of the junction. The protein has a second function as a scaffolding protein for the GTPase RhoA and associated Rho regulatory proteins (18,19). In addition, it can also directly interact with the zinc finger transcriptional repressor Kaiso (ZBTB33), facilitating Wnt signal transduction (20,21). Thus, p120-catenin appears to be a multi-functional protein, promoting epithelial stability when in complex with E-cadherin and regulating RhoA and transcriptional activities. p120-catenin is also able to associate with mesenchymal cadherins such as N-cadherin and cadherin-11 (17,22). In mesenchymal cells, p120-catenin associates with non-epithelial cadherins, regulating motility and invasion via cytoskeletal events and transcription. Given its functions in both epithelia and mesenchyme, it is unsurprising that both the loss and gain of p120-catenin have been associated with oncogenesis (23–25).

In humans, the *CTNND1* gene is located at 11q11 and consists of 21 exons; of which, exons 11, 18 and 20 are alternatively spliced. Inclusion of exon 11, which is predominantly neural, disrupts a nuclear localization signal, while exon 20 contains a nuclear export signal (26). In addition, there are four additional isoforms of the protein, which vary in their transcriptional start sites. Of the four major isoforms, isoform 1 is abundant in mesenchymal cells, while isoform 3 appears preferentially expressed in epithelial cells (27–30). The other two isoforms are less well characterized.

The p120 superfamily includes p120-catenin itself, δ-catenin (CTNND2) and ARVCF [armadillo repeat gene deleted in velocardiofacial (VCF) syndrome] all of which can compete for E-cadherin binding. Although it is unclear whether they substitute for one another in other cellular functions (31,32), evidence from animal studies suggests some compensatory roles. For instance, δ-catenin (CTNND2) knockdown phenotypes can be rescued with p120-catenin, and the combined depletion of δ-catenin and p120 generates more pronounced effects. However, levels of p120 are not altered by reducing δ-catenin protein levels (33). In humans, *CTNND2* variants have been associated with autism spectrum disorders and other neurodevelopmental conditions (34–39). Interestingly, the other p120 family member, *ARVCF*, lies in 22q11. While the loss of *TBX1* in 22q11 is thought to cause the key malformations associated with VCF syndrome [MIM: 192430], evidence from animal models suggests that *ARVCF* may also play a role in craniofacial development (40–43).

Here, we present a multi-system condition beyond that described in known p120-associated cases, which was recently described in the context of BCD (1–3). However, the majority of reported BCD cases are caused by E-cadherin variation (1–3). While our subjects possess palatal phenotypes and eyelid anomalies, they also consistently display additional features including cardiac, limb and neurodevelopmental anomalies. Only a subset of our participants had eyelid symptoms, and none were diagnosed with BCD prior to genetic analysis. Therefore, we propose that these novel truncating variants in *CTNND1* should be considered to be a phenotypic expansion beyond BCD. Furthermore, we propose that these variants affect both E-cadherin-dependent and -independent functions of p120-catenin and, given the range of phenotypes seen in our cohort, should be considered more broadly to cause a VCF-like syndrome.

## Subjects and Methods

### Recruitment, consent and sample collection

Participants were recruited from one of following: South Thames Cleft Unit at Guy’s and St Thomas Trust (GSTT), London, UK; theUniversity of Calgary, Alberta Children’s Hospital, Canada; the Children’s Hospital of Philadelphia, USA; or the Deciphering Developmental Disorders (DDD) Study, UK (www.ddduk.org). *CTNND1* data access was specifically collected under DDD Project CAP180, focusing on cranial neural crest anomalies (ABM/KJL). All individual study protocols were approved by local Institutional Review Boards, including UK Ethics: GSTT (REC16/NI/0026, Northern Ireland REC) and DDD (10/H0305/83, Cambridge South REC, and GEN/284/12, Republic of Ireland REC).

Medical and dental histories were taken, as well as detailed phenotyping by clinical geneticists with expertise in dysmorphology. Saliva for DNA extraction was collected from family trios using the Oragene® DNA (OG-500) kit. All patients also underwent high-resolution analysis for copy number abnormalities using array-based comparative genomic hybridization. Informed consent from all participants was obtained for the publication of data and photographs in the medical literature. All families were offered genetic counseling.

### Whole exome sequencing and variant screening

Whole exome sequencing (WES) from trios was performed to identify gene variants. For patients recruited from DDD (44), genomic DNA samples from trios were analysed at the Wellcome Trust Sanger Institute. WES was performed using a custom Agilent SureSelect Exome bait design (Agilent Human All Exon V3 Plus with custom ELID # C0338371), 8-plex sample multiplexing and an Illumina HiSeq with four samples per lane and a mean depth of 50X. The exome analysis targeted 58.62 Mb of which 51.64 Mb consisted of exonic targets (39 Mb) and their flanking regions and 6.9 Mb consisted of regulatory regions. Alignment was performed using BWA1. Putative *de novo* variants were identified from trio BAM files using DeNovoGear5. Variants were annotated with the most severe consequence predicted by Ensembl Variant Effect Predictor (VEP version 2.6), and minor allele frequencies from a combination of the 1000 Genomes project (www.1000genomes.org), UK10K (www.uk10k.org), the NHLBI Exome Sequencing Project (esp.gs.washington.edu), Scottish Family Health Study (www.generationscotland.org), UK Blood Service and unaffected DDD parents. All flagged variants were automatically annotated with pathogenicity scores from two variant prioritization algorithms (SIFT23 and PolyPhen24) and compared against the public Human Gene Mutation Database and the Leiden Open Variation Database. For selected probands, WES performed at the Yale Center for Genomic Analysis used genomic DNA isolated from saliva from the probands and their parents. The exons and their flanking regions of the genome were captured using IDT xGen exome capture kit followed by Illumina DNA sequencing (HiSeq 4000). Paired end sequence reads were converted to FASTQ format and were aligned to the reference human genome (hg19). GATK best practices were applied to identify genetic variants, and variants were annotated by ANNOVAR. Probands and parents were sequenced to a mean depth of 93–123 independent reads per targeted base across all the samples. In an average of 94.0% of targeted bases in all of the samples, the coverage was greater than 20X independent reads. Trio WES analysis on variants with allele frequency of less than 1% was carried out to identify *de novo* variants that are absent from the parents. Putative disease-causing variants were validated using whole genome amplified DNA, PCR and capillary sequencing.

### Mouse and *Xenopus* husbandry

Animal work was performed in accordance with UK Home Office Project License P8D5E2773 at King’s College London (KJL), University of Texas Southwestern Medical Center Institutional Animal Care and Use Committee protocols (DKM), the European *Xenopus* Resource Centre, Portsmouth UK, or the Yale University Institutional Animal Care and Use Committee protocols (MKK). Mice were genotyped according to standard procedures. Gestational ages for mice were determined by the observation of vaginal plugs, which was considered embryonic day 0.5 (E0.5) and further staging of animals according to Kaufman (45). The following mouse strains were used: *Ctnnd1^fl/fl^* (MGI ID: 3640772) (8); *β-actin::cre* (JAX strain 019099) (46) and *Wnt1::cre* (JAX strain 022501) (47)*.* For each mouse experiment, a minimum of *n* = 3 was examined unless otherwise noted. *Xenopus tropicalis* embryos were produced by *in vitro* fertilization and raised to appropriate stages in 1/9MR + gentamycin as per standard protocols (48). For *Xenopus* experiments, experimental numbers are stated in figures, with a minimum of *n* = 30 in all experimental conditions.

### Human specimens

Human embryonic and fetal material was provided by the Joint MRC/Wellcome Trust (Grant #099175/Z/12/Z) Human Developmental Biology Resource (HDBR, http://www.hdbr.org) as whole embryos [Carnegie stage 13 (C13, day 28–32)] or sectioned embryos [Carnegie stage 21 (C21, day 50–52)].

### Generation of *CTNND1* probe and mRNA *in situ* hybridization

A human *CTNND1* clone was identified from the Human ORFeome Collaboration (49) (clone HsCD00513511), encoding *CTNND1* isoform 4, including the entirety of the armadillo repeats and the C-terminal domain. Probes made from this clone should recognize all four *CTNND1* transcripts. Digoxigenin-labeled antisense mRNA probes were produced by linearizing human *CTNND1* clones using BamH1 restriction enzyme, which produces a probe size of ~ 900 base pairs, and *in vitro* transcription with the T7 High Yield RNA Synthesis Kit (E2040S) from New England Biolabs. *In situ* hybridization of mRNA on whole mount and paraffin embedded tissue sections was carried out as per standard protocols (50), using an anti-digoxigenin-alkaline phosphatase coupled antibody.

### Immunofluorescent antibodies and staining

For immunostaining, mouse embryos at the indicated stages were fixed and processed according to standard protocols. Antigen retrieval was carried out in Tris-EDTA (pH 9) in a 90°C water-bath for 30 min. Primary antibodies used were: phospho-tyrosine p120-catenin clone 2B12, mouse mAb (1:150, Biolegend, Cat. No. 828301); delta 1 Catenin/CAS (phospho S-268) antibody [EPR2380], rabbit mAB (1:150, Abcam, Cat. No. ab79545); E-Cadherin [M168], mouse mAB (1:150, Abcam, Cat. No. ab76055); anti-E-cadherin (24E10), rabbit mAb (1:250, Cell Signaling Technology, Cat. No. 3195); rabbit anti-Pax2 Antibody (1:100, ThermoFisher Scientific, Cat. No. 71-6000) and mouse anti-Collagen Type II, clone 6B3 (1:50, MERCK, Cat. No. MAB8887). Secondary antibodies used were: Alexa Fluor® 488 (Invitrogen, A-11008), Alexa Fluor® 488 (Invitrogen, A-21204), Alexa Fluor® 546 (Invitrogen, A-11060), Alexa Fluor® 568 (Invitrogen, A-11011), Alexa Fluor® 594 (Invitrogen, A-21207) and Alexa Fluor® 647 (Invitrogen, A-21235). All were diluted to 1:400 in phosphate-buffered saline (PBS) containing 0.5% Triton® X-100 (Sigma-Aldrich) and 1% bovine serum albumin. Slides were mounted in Fluoroshield Mounting Medium with DAPI (Abcam, ab104139) and cover slipped. *Xenopus* whole mount embryos and tadpoles were incubated with Hoechst (1:5000 of 20 mg/ml, diluted in PBST). For hematoxylin and eosin (H&E) staining, slides were fixed, sectioned and stained according to standard protocols. Slides were then cover slipped with Neo-Mount (VWR, Cat. No. 1.09016.0500).

### Image acquisition

Images for sectional *in situ* hybridization experiments and for H&E slides were captured using a brightfield microscope (Nikon ECLIPSE Ci-L), with an attached camera (Nikon digital sight DS-Fi1) or with a NanoZoomer 2.ORS Digital Slide Scanner (Hamamatsu); NDP.view2 Viewing Software (U12388-01) was used to analyze the scanned images. Whole mount images of mouse pups and embryos, *Xenopus* and human embryos were captured using a Nikon SMZ1500 stereomicroscope with a Nikon digital sight DS-Fi1 (112031) camera. Fluorescent images of mouse palates and *Xenopus* epithelial cells were either acquired on a Leica SP5 confocal or Nikon A1R point scanning confocal; z-stacks of whole mount *Xenopus* tadpoles were captured by mounting the tadpoles on a Cellview Cell Glass Bottom Culture Dish (PS, 35/10 mm, CELLview™, Cat. No. 627860) in PBS. Image sequences were processed using the FIJI (Image J) analysis software.

### Micro-computed tomography

For soft tissue scanning, mouse embryos were stained with a near isotonic 1% I2 and 2% potassium iodine solution for 3 days and scanned to produce 6 μm voxel size volumes, using X-ray settings of 90 kVp, 66 uA and a 0.5 mm aluminium filter to attenuate harder X-rays. Camera binning was used to improve signal-to-noise ratios. For hard tissue staining, perinatal mice were scanned to produce 7.4 μm voxel size volumes using X-ray settings of 70 kVp, 114 uA and a 0.5 mm aluminium filter to attenuate harder X-rays. The specimens were analysed using Parallax Microview software package (Parallax Innovations Inc., Ilderton, ON, Canada). Specimens were scanned using a Scanco μCT50 microcomputed tomographic (μCT) scanner (Scanco, Brüttisellen, Switzerland). The specimens were immobilised in appropriately sized scanning tubes using cotton gauze.

### CRISPR/Cas9 knockouts in *X. tropicalis*

The following non-overlapping single guide RNAs (sgRNAs) were designed to target *X. tropicalis ctnnd1*: sgRNA1—CTAGCtaatacgactcactataGGAACGGGTGTGGGAGCCATgttttagagctagaa and sgRNA2—CTAGCtaatacgactcactataGGGGTGGTATCCCACGCAAGgttttagagctagaa. sgRNA1 targets exon 3 and is thus predicted to disrupt isoform 1 only, while sgRNA2 targets exon 7 and is thus predicted to disrupt all four isoforms. Embryos were injected at the one- or two-cell stage and raised until indicated stages. For CRISPR/Cas9 experiments, statistical significance was defined as *P* < 0.05 and analysed by chi-squared test or Fisher’s exact test.

**Figure 1:**
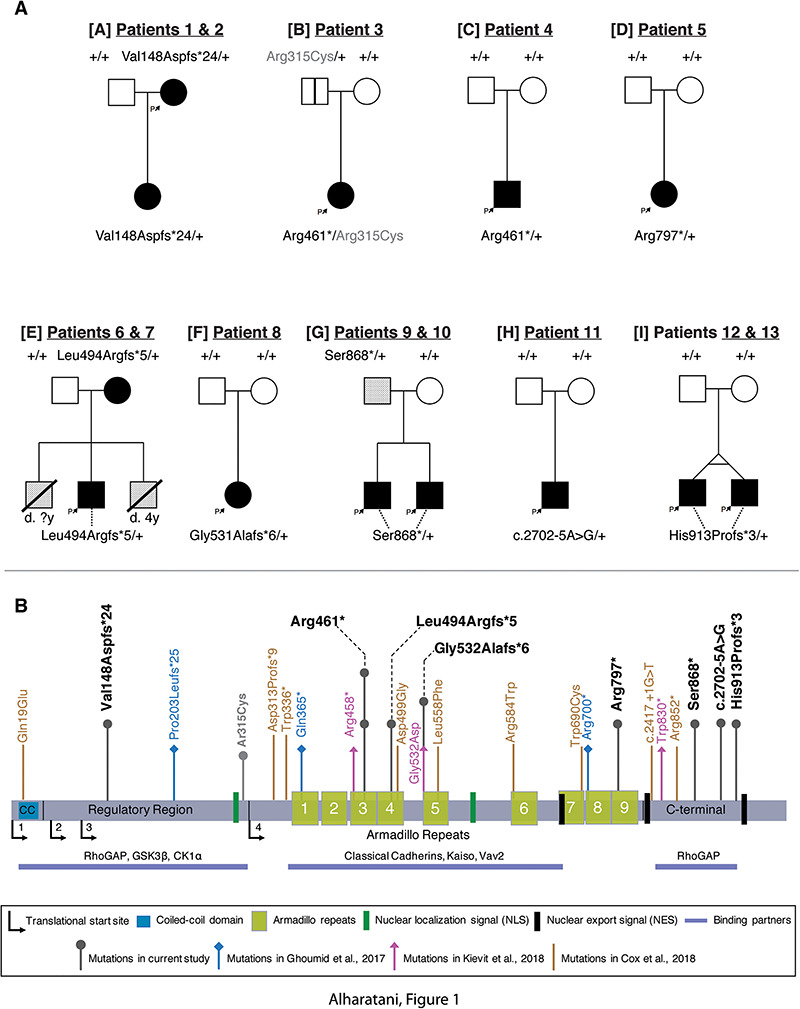
Pedigrees and identification of CTNND1 variants. (**A**) Pedigrees of individuals with identified variants. Family identifications, in brackets [A-I], and patient identification numbers correlate with Table 1. Filled boxes indicate affected individuals demonstrating collective phenotypes described in our cohort. A blank box with a vertical black line indicates an asymptomatic carrier (clinically unaffected). A box with an oblique line indicates a deceased individual. Lightly shaded boxes indicate individuals affected with one or more of the conditions described. (**B**) Schematic representation of the human p120-catenin protein structure and its domains. The variants described in our cohort are shown above the protein with a dark gray arrow. The light gray arrow with the (p.Arg315Cys) variant indicates the other *CTNND1* mutation found in Patient 3 which was inherited from the unaffected father [A]. Arrows in blue, pink and brown represent the variants and their locations reported in Ghoumid *et al*. (2), Kievit *et al*. (1) and Cox *et al*. (3), respectively.

## Results

### Identification of *CTNND1* variants

Here, we identify 13 individuals from nine families with novel protein-truncating variants in *CTNND1*. These mutations were not previously described in BCD, orofacial cleft cases nor in gnomAD (Table 1). Family trees are shown in Figure 1A, while mutations from this study and from previous studies have been mapped onto a protein schematic (Fig. 1B). Notably, two unrelated individuals had the same novel (p.Arg461^*^) variant we describe here (Fig. 1). Previously, all subjects had undergone an array-based comparative genome hybridization analysis with normal results. A subset of participants had also been referred for other diagnostic tests, including 22q11 deletion, Down syndrome, CHARGE syndrome (*CHD7* sequencing), Noonan syndrome (*PTPN11* sequencing) and other conditions, but with no definitive diagnoses. WES of the nine families revealed eight novel variants in *CTNND1*, including six confirmed to have arisen *de novo* (in eight patients). Two individuals inherited their variant from affected parents, while two other participants inherited a variant from a parent with a mild phenotype (Fig. 1A). These truncating mutations included nonsense, splicing and frameshift variants (Table 1).

**Table 1:** *CTNND1* variants in index patients

Family	Patient	Mutation: NM_00108558.1	Protein: NP_001078927.1	Variant type	Exon	Inheritance	gnomAD
A	1	c.443_444delTG	p.Val148Aspfs^*^24	Frameshift	6	*De novo*	Novel
	2	c.443_444delTG	p.Val148Aspfs^*^24	Frameshift	6	Maternally inherited	Novel
B	3	c.943C>T	p.Arg315Cys	Missense	6	Paternal, unlikely causal	2.44 e − 4 8 FE, 39 NFE, 4 A
	3	c.1381C>T	p.Arg461^*^	Nonsense	7	*De novo*	Novel
C	4	c.1381C>T	p.Arg461^*^	Nonsense	7	*De novo*	Novel
D	5	c.2389C>T	p.Arg797^*^	Nonsense	15	*De novo*	Novel
E	6	c.1481_1485del	p.Leu494Argfs^*^5	Frameshift	8	Not determined	Novel
	7	c.1481_1485del	p.Leu494Argfs^*^5	Frameshift	8	Maternally inherited	Novel
F	8	c.1595del	p.Gly532Alafs^*^6	Frameshift	8	*De novo*	Novel
G	9	c.2598_2601dupTGAT	p.Ser868^*^	Nonsense	17	Paternally inherited	Novel
	10	c.2598_2601dupTGAT	p.Ser868^*^	Nonsense	17	Paternally inherited	Novel
H	11	c.2702-5A>G	p.?	Splice site	18–19	*De novo*	Novel
I	12	c.2737dupC	p.His913Profs^*^3	Frameshift	19	*De novo*	Novel
	13	c.2737dupC	p.His913Profs^*^3	Frameshift	19	*De novo*	Novel

The Human GRCh37 (hg19) Assembly was used to identify transcript positions. The annotations are all based on the NM_001085458 transcript. Asterisk denotes termination codon; FE, Finnish European; NFE, non-Finnish European; A, African.

*CTNND1* variants identified could be grouped according to the overall structure of the protein (Fig. 1B). Beginning with the N-terminal regulatory region, one variant falling within this region was identified in a mother (Patient 1) and later confirmed in her affected daughter by targeted sequencing (Patient 2). The mother’s *de novo CTNND1* variant is designated as c.443_444delTG (p.Val148Aspfs^*^24) affecting exon 6.

Four variants fell within the armadillo repeats, which are predicted to be crucial for interactions with E-cadherin. Two unrelated individuals (Patients 3 and 4) from two different families had the same *de novo* mutation in *CTNND1:* c.1381C>T (p.Arg461^*^) (Fig. 1A and B). This variant results in a non-sense substitution and creates a stop codon in exon 7. In addition, Patient 3 had a rare variant in *CTNND1*, inherited paternally c.943C>T (p.Arg315Cys), which is present at a frequency of 2 × 10^–4^ in reference populations (51). As the parent shares none of the phenotypes with the patient, this second variant is unlikely to be causative. Moreover, a *CTNND1* frameshift variant c.1481_1485del (p.Leu494Argfs^*^5) in exon 8 was identified in a mother and child; both are affected (Patients 6 and 7, respectively); however, the *de novo* status in the mother had not been confirmed. In the same exon, Patient 8 had a *de novo CTNND1* variant designated as c.1594del (p.Gly532Alafs^*^6). Finally, a *de novo* mutation in armadillo 9 was found in Patient 5 designated as c.2389C>T (p.Arg797^*^) on exon 15.

We found three variants affecting the C-terminal domain, which were present in five patients in three families. The variant c.2598_2601dupTGAT (p.Ser868^*^) was paternally inherited in a family with two affected siblings (Patients 9 and 10). Anecdotally, the father is fit and healthy; however, his palate is narrow and high, and his nose is prominent; his *de novo* status has not been confirmed. Patient 11 has a *de novo CTNND1* variant at the splice acceptor site of exon 19 designated as c.2702-5A>G, which is predicted to create a cryptic splice site, leading to a premature termination codon at the start of exon 19. Finally, Patients 12 and 13 are monozygotic twins carrying a *de novo* frameshift variant in *CTNND1*: c.2737dupC (p.His913Profs^*^3).

### Clinical presentation of patients with *CTNND1* variants

**Figure 2:**
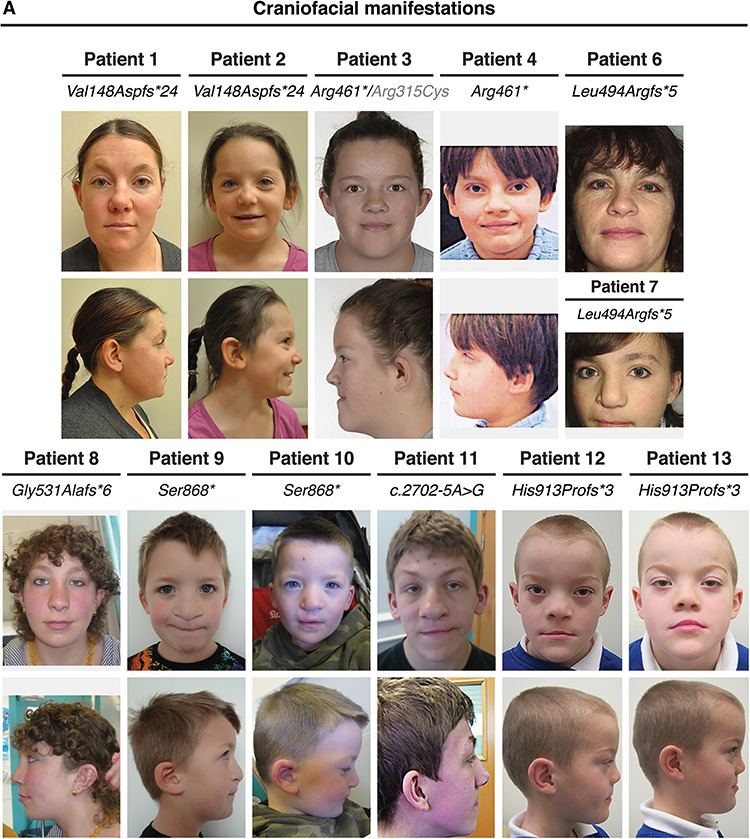
Clinical presentation of individuals with a CTNND1 mutation. Facial photos (frontal and profile) show craniofacial features of patients. Note the narrow up-slanting palpebral fissures in Patients 3, 4 and 7–13; the hooded eyelids in Patients 3, 4 and 8–13; telecanthus in Patients 3, 4 and 9–13; the high arched eyebrows in Patients 1, 2, 6–8 and 11–13 and the thin lateral eyebrows in Patients 1 and 5–11. Patients 1 and 4 had missing eyelashes medially from the inner canthus; Patients 1, 2, 5 and 7 have distichiasis (double row of lashes), and mild ectropion of the lower eyelids was seen in Patients 1, 5 and 6. As evident, no patient shows signs of hair sparsity. Most patients had wide nasal bridges with broad nasal tips, while Patients 1, 2, 8 and 11 were also diagnosed with congenital choanal atresia. Patients 1, 2, 7–9, 11 and 12 showed thin upper lips, and while mid-face hypoplasia was observed, Patients 1, 3, 8, 11 and 13 also had mandibular prognathism. Scars from cleft lip operations are seen in Patients 7 and 9–13. Patient 3 was born with a sub-mucous cleft palate, a bifid uvula and VPI.

**Figure 3:**
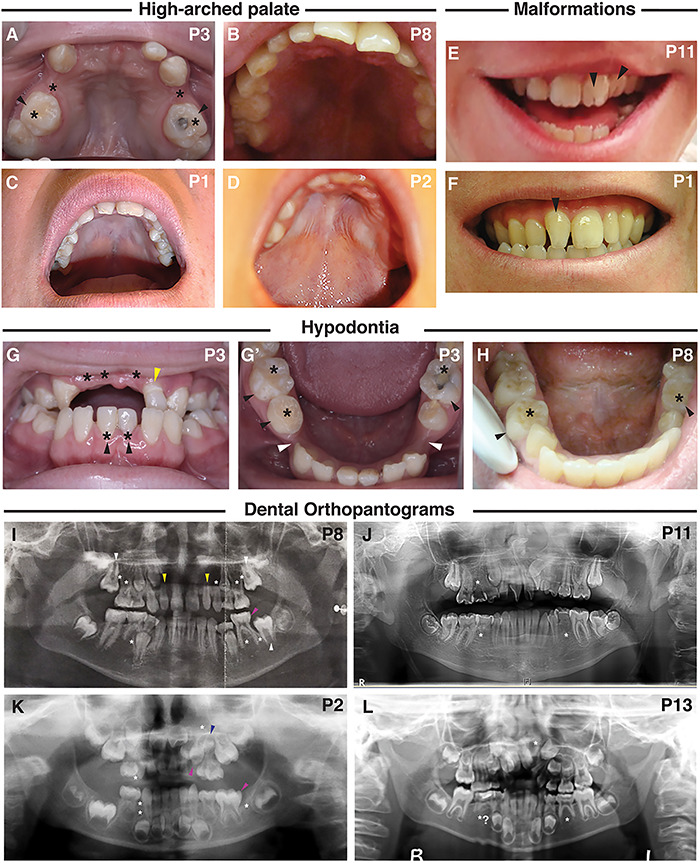
Dental manifestations and intra-oral phenotypes of patients with a CTNND1 mutation. (**A**–**D**) A high-arched palate was seen, shown are palates of Patients 1, 2, 3 and 8. (**E**, **F**) Abnormalities in the morphology of the dentition included: fissured incisors in patient 11 (E, black arrowheads) and rotation of the incisors from the normal alignment shown in the non-cleft Patient 1 (F, black arrowhead). (**G**, **H**) Hypodontia (tooth agenesis) was a common phenotype, indicated by the black asterisk. Black arrowheads indicate retained primary teeth. Patient 3 also has a diminutive upper left lateral incisor (G, yellow arrowhead) and wide inter-dental spacing (G′, white arrowheads). (**I**–**L**) Dental orthopantograms (OPGs); missing teeth are indicated by white asterisks; diminutive teeth by yellow, macrodont teeth by magenta and supernumerary teeth by blue arrowheads, respectively. (I) OPG of Patient 8 at age 11 shows eight missing permanent teeth (white asterisks) and shows the eruption of the second permanent molars (white arrowheads) in place of the missing first permanent molars. Also shown are diminutive upper right and left lateral incisors (peg-shaped) (yellow arrowheads) and a macrodont lower left second primary molar (magenta arrowhead). (J) OPG of Patient 11, at the age of 14, shows three missing permanent teeth (white asterisks), an ectopic maxillary left permanent canine and rotated maxillary centrals and left lateral incisors and dilacerated roots of the lower second permanent molars. (K) OPG of Patient 2, taken at 4 years, shows missing teeth including a missing lower left first permanent molar (white asterisks); a reported macrodont upper left primary canine (magenta arrowhead) with an underlying missing successor (white asterisk); a macrodont lower left second primary molar (magenta arrowhead) and a supernumerary tooth (blue arrowhead). (L) OPG for Patient 13, taken at 7.5 years, confirms the absence of the upper left permanent lateral incisor and possibly the lower second permanent premolars.

Clinical phenotypes are summarized in Table 2, and further details can be found in Supplementary Material, Table S1. Also included in Supplementary Material, Table S1 are the summaries of phenotypes seen in previous reports (1,2). Photographs from participants show a number of shared craniofacial and oral features (Figs 2 and 3, respectively) as well as other affected structures [eyes, ears and limbs (Supplementary Material, Figure S1)]. Additional features including heart anomalies and neurodevelopmental conditions are noted in Table 2 and Supplementary Material, Table S1.

**Table 2:** Clinical summary of individuals with *CTNND1* variants

Participants	1	2	3	4	5	6	7	8	9	10	11	12	13	Total
Variant	V148Dfs^*^24	V148D^*^24	R461^*^	R461^*^	R797^*^	L494Rfs^*^5	L494Rfs^*^5	G531Afs^*^6	S868^*^	S868^*^	c.2702-5A>G	H913Pfs^*^3	H913Pfs^*^3	−
Sex	F	F	F	M	F	F	M	F	M	M	M	M	M	6F/7M
Craniofacial														
Cleft lip/palate	−	−	+	−	−	+	+	−	+	+	+	+	+	8/13
High-arched palate	+	+	+	−	−	−	+	+	−	ND	−	+	+	7/13
Thin upper lip	+	+	−	−	−	−	+	+	+	−	+	+	−	7/13
Choanal atresia	+	+	−	−	−	−	−	+	−	−	+	−	−	4/13
Ear anomaly	−	+	+	+	−	+	+	+	+	+	+	−	−	9/13
Wide nasal bridge	+	+	+	−	−	+	+	+	+	+	+	+	+	11/13
Broad nasal tip	+	−	+	−	−	−	+	+	+	+	+	−	−	7/13
Mid-facial hypoplasia	+	+	+	−	−	+	+	+	−	−	+	+	+	9/13
Mandibular prognathism	+	−	+	−	−	−	−	+	−	−	+	−	+	5/13
Brachycephaly	−	+	−	+	−	−	−	−	−	−	+	−	−	3/13
Eyes and eyelids														
Narrow, upslanted palpebral fissures	−	−	+	+	−	−	+	+	+	+	+	+	+	9/13
Hooded eyelids	−	−	+	+	−	−	−	+	+	+	+	+	+	8/13
Telecanthus	−	−	+	+	−	−	−	−	+	+	+	+	+	7/13
High arched eyebrows	+	+	−	−	−	+	+	+	−	−	+	+	+	8/13
Thin lateral eyebrows	+	−	−	−	+	+	+	+	+	+	+	−	−	8/13
Mild ectropion	+	−	−	+	+	+	−	−	−	−	−	−	−	4/13
Distichiasis	+	+	−	−	+	−	+	−	−	−	−	−	−	4/13
Ankyloblepahron	−	+	−	−	−	−	+	−	+	−	−	−	−	3/13
Dental anomalies														
Hypodontia	+	+	+	+	+	−	−	+	ND	ND	+	−	+	8/13
Delayed dentition	+	+	−	+	+	−	−	ND	ND	ND	+	−	+	6/13
Abnormal crown form	+	+	+	−	+	−	+	+	+	ND	+	+	−	9/13
Cardiac disease														
VSd	+	+	−	+	−	−	−	−	−	−	+	+	−	Total 6/13
TOF	−	−	−	−	−	−	−	+	−	−	−	−	−	
ASd or PFO	+	+	−	+	−	−	−	−	−	−	−	−	−	
MVS	+	−	−	−	−	−	−	−	−	−	−	−	−	
PS or COA	−	−	−	−	−	−	−	−	−	−	+	−	−	
PDA	−	+	−	−	−	−	−	−	−	−	−	−	−	
Hypoplastic aortic arch	+	−	−	−	−	−	−	−	−	−	+	−	−	
Neurodevelopmental														
ASD	−	UI	+	+	−	−	−	−	UI	−	+	−	−	Total 8/13
ADHD	−	+	+	−	−	−	−	−	−	−	+	−	−	
DD/LD	−	+	+	−	−	−	−	−	−	+	+	+	+	
Speech & language delay	−	−	+	−	−	−	−	−	−	+	+	−	−	
Aggressive behaviour	−	+	+	−	−	−	−	−	+	+	−	−	−	
Limb anomalies														Total 9/13
Hands	−	−	+	−	−	+	+	+	−	−	+	+	+	7/13
Feet	−	+	+	+	−	+	+	−	−	−	−	+	+	7/13
Voice anomalies	−	+	−	−	ND	+	−	−	−	−	+	−	−	3/13
Other Skeletal	+	−	+	+	−	+	−	−	−	−	+	−	−	Total 5/13
Scoliosis	+	−	−	−	−	+	−	−	−	−	−	−	−	
Short stature	−	−	−	−	−	+	−	−	−	−	+	−	−	
Cancer	−	−	−	−	−	−	−	ovarian dysgerminoma	−	−	−	−	−	1/13
Other anomalies	restrictive lung disease	partial agenesis of corpus callosum	VPI, early onset puberty, bowel problems	joint laxity	−	hypothyroid	−	macroglossia	−	−	cryptorchidism	coronal hypospadias	−	−

Abbreviation: UI, under investigation, ND; not determined because of non-availability; VSd, ventricular septal defect; ASd, atrial septal defect; TOF, tetralogy of Fallot; CoA, coarctation of the aorta; MVS, mitral valve stenosis; PDA, patent ductus arteriosus; PFO, patent foramen ovale; ASD, autism spectrum disorder; ADHD, attention deficit hyperactivity disorder; DD, developmental delay; LD, learning difficulty; VPI, velo-pharyngeal insufficiency.

Participants shared several distinctive eye features including short, up-slanted palpebral fissures (9/13), hooded eyelids (8/13), telecanthus (7/13), highly arched (8/13) and thin lateral eyebrows (8/13) and other eyelid anomalies such as nasolacrimal obstructions (1/13). These eye anomalies were clear from a young age (Supplementary Material, Figure S1A). A subset had ectropion (drooping lower eyelids, 4/13) and distichiasis (double eyelashes, 4/13). Many individuals had wide nasal bridges (11/13) with broad nasal tips (7/13), choanal atresia (4/13), either unilateral or bilateral atresia; malar flattening (mid-face hypoplasia) (9/13); mandibular prognathism (5/13); thin upper lips (7/13) and auricular abnormalities (9/13), particularly low-set ears and overfolded helices (Supplementary Material, Figure S1B).

Phenotypes with high penetrance involved oropharyngeal abnormalities including cleft lip and/or palate (CLP) (8/13), high-arched palate (7/13) or a combination of cleft and high-arched palate (Fig 3A–D). A range of cleft sub-types was seen (Supplementary Material, Table S1). In addition, one participant had velopharyngeal insufficiency (VPI) and a bifid uvula. Of interest, three individuals presented with vocalization defects causing stridor and hoarseness or nasal speech.

Upon dental examination, all subjects were found to have intra-oral anomalies (Fig. 3). In particular, congenital tooth agenesis (hypodontia) was frequently seen, with eight subjects missing between 3 and 12 adult teeth (Fig. 3G–L; Supplementary Material, Table S2). Other anomalies included retained primary teeth and delayed eruption of the permanent teeth (6/13) (Supplementary Material, Table S1). Morphologic tooth anomalies were present, including diminutive permanent teeth/peg-shaped lateral incisors and fissured crowns of the permanent central and lateral incisors (Fig. 3E and F; Supplementary Material, Table S1).

Beyond the craniofacial structures, the majority of the participants had limb and heart anomalies. Mild limb phenotypes (9/13) were present, including shorter fifth fingers, single transverse palmar crease, mild syndactyly between the 2 and 3 toes, sandal gaps and camptodactyly of the toes (Supplementary Material, Figure S1C). Congenital cardiac defects, which have not previously been associated with *CTNND1* variants, consistently occurred in our cohort. Six subjects had cardiovascular anomalies including tetralogy of Fallot, hypoplastic aortic arch, coarctation of the aorta, ventricular septal defect, atrial septal defect, mitral valve stenosis, patent ductus arteriosus and patent foramen ovale (Table 2; Supplementary Material, Table S1). Finally, in addition to the craniofacial and cardiac anomalies, individuals presented with other phenotypes that added to the complexity of their conditions. Developmental delay and other neurodevelopmental problems were also observed (8/13). These often appeared from early toddler and school years and included mild learning difficulties, autism spectrum disorder, speech and language delay and behavioral problems (Supplementary Material, Table S1). One individual was diagnosed with ovarian dysgerminoma stage III in the left ovary at the age of 12 years, which was treated with left oophorectomy followed by chemotherapy. Other infrequent anomalies included urogenital problems, scoliosis and partial agenesis of the corpus callosum (Supplementary Material, Table S1).

### *CTNND1* is expressed during human embryonic development

*CTNND1* has recently been linked to non-syndromic cleft palate (3). In that study, Cox *et al*. (3) documented the protein distribution of human p120-catenin protein, focusing on the fusion of the secondary palate. However, to our knowledge, little is known about human *CTNND1* mRNA expression during pharyngeal arch stages. Given the multi-system anomalies seen in our subjects, it was important to examine expression at earlier stages during the development of the cranio-cardiac structures. Therefore, we carried out mRNA *in situ* hybridization on human embryos using a probe that binds to all four C*TNND1* mRNA transcripts.

**Figure 4:**
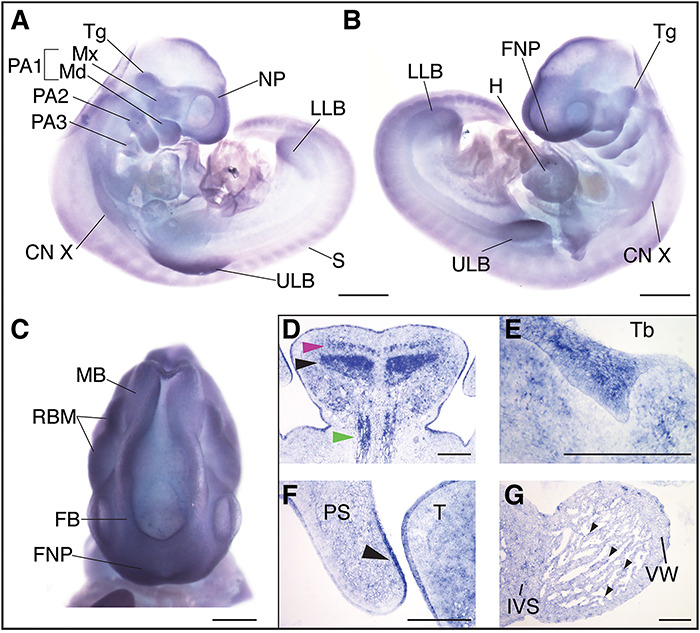
P120-catenin is expressed during relevant stages of human embryonic development. *CTNND1* mRNA *in situ* hybridization at human Carnegie stages 13 (CS13) (**A**–**C**) and 21 (**D**–**G**). (A) Right lateral view of a CS13 human embryo, *CTNND1* mRNA is strongly expressed in the head in all three pharyngeal arches (PA1, PA2 and PA3) and limb buds. Expression is specifically strong around the nasal placode and the maxillary and mandibular prominences. (B) Left lateral view, P120 is strongly expressed in the developing heart, frontonasal process, the trigeminal ganglion and the tenth cranial nerve. (C) P120 is ubiquitously expressed in the developing brain region in the rhombomeres, the forebrain and midbrain. (D–G) Coronal section through the head of a CS21 human embryo through a mid-palatal plane. (D) Strong expression is seen in the intrinsic muscles of the tongue: the superior longitudinal (magenta arrowhead), the transversal muscles of the tongue (black arrowhead) and the extrinsic genioglossus muscle (blue arrowhead). (E) *CTNND1* mRNA is strongly expressed in the epithelium of the developing tooth bud. (F) *CTNND1* is expressed on the dorsal epithelium of the palatal shelf (arrowhead) and in the epithelium of the tongue. (G) Expression is seen in the cardiomyocytes of the ventricular wall and the interventricular septum and in the cells of the endocardium (arrowhead). Scale bars = 100 μm. Abbreviations: PA1, first pharyngeal arch; PA2, second pharyngeal arch; PA3, third pharyngeal arch; Tg, trigeminal ganglion; Mx, maxillary process; Md, mandibular process; CN X, tenth cranial nerve; ULB, upper limb bud; S, somites; LLB, lower limb bud; NP, nasal placode; H, heart, FNP, frontonasal process; Tb, mandibular tooth bud; PS, palatal shelf; T, tongue; IVS, interventricular septum; VW, ventricular wall.

At Carnegie stage 13 (CS13), we found expression at multiple sites within the developing head, including the frontonasal processes, the forebrain, midbrain and rhombomeres (Fig. 4B and C). Robust expression was also detected in the maxillary and mandibular processes of the first pharyngeal arch (PA1), the second and third pharyngeal arches (PA2 and PA3, respectively) as well as in the proximal domains of the upper and lower limb buds (Fig. 4A and B). Signal was also weakly detected in the somites; however, strong expression was seen in the developing heart, trigeminal ganglion and the 10th cranial nerve (Fig. 4A and B).

By Carnegie stage 21, *CTNND1* mRNA was expressed in the brain (data not shown), tooth bud (Fig. 4E), the epithelial lining of the tongue and oral cavity and in the tongue mesenchyme (Fig. 4D). Expression was particularly strong in the intrinsic muscles of the tongue: the superior longitudinal and transversal muscles, and in the extrinsic genioglossus muscle (Fig. 4D). Moreover, expression was evident in the dorsal epithelial lining of the developing palatal shelves (Fig. 4F). In the heart, *P120* expression was found in the cardiomyocytes of the ventricular wall and interventricular septum, in addition to strong expression in the endocardium (Fig. 4G). Expression was also found in the intrinsic epithelial lining of the stomach wall; both in the pyloric part of the stomach and in the inner walls of the stomach body, the pancreatic islets, the germinal center of the spleen, the epithelial lining of the bladder, hindgut and in the spinal cord and vertebral body (Supplementary Material, Figure S2).

### Expression of phosphorylated p120-catenin predicts fusion of the palatal seam

**Figure 5:**
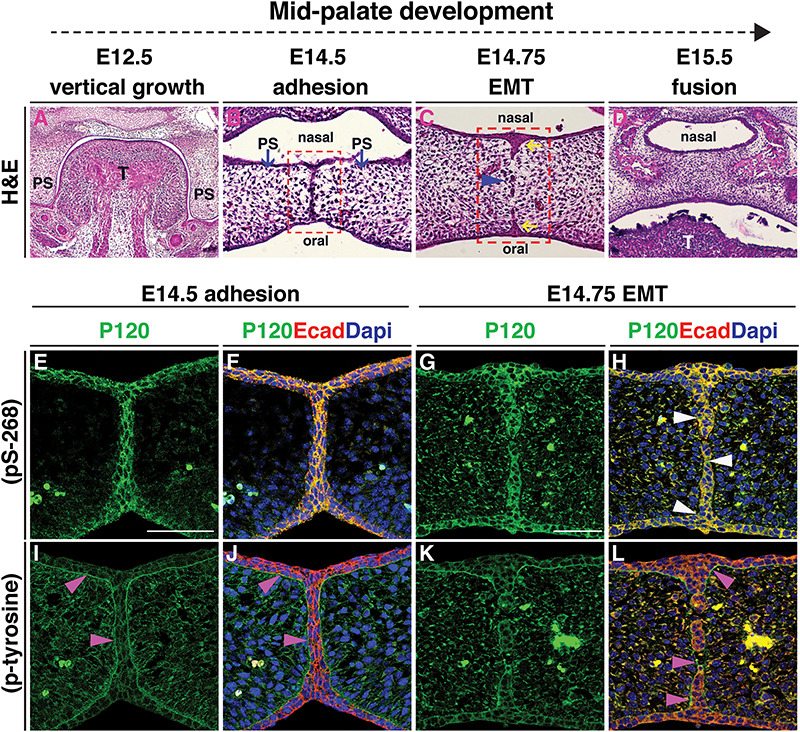
Expression of phosphorylated p120-catenin predicts fusion of the palatal seam. (**A**–**L**) All images are coronal sections of CD1 wild-type murine embryos at consecutive stages of palatal development. (A–D) H&E staining illustrates successive stages of palatogenesis from embryonic day (E) 12.5 to E15.5. (B) At E14.5, following horizontal elevation, the opposing palatal shelves (blue arrows) meet and adhere to form the MES. (C) EMT occurs at E14.75 when the MES breaks down, forming epithelial islands (blue arrowhead); the nasal and oral epithelial triangles form (yellow arrows). (D) At E15.5 palatal shelves are fused. Red box in (B) marks the regions shown in (E, F, I and J). Red box in (C) marks the regions shown in (G, H, K and L). (E–L) Immunofluorescent staining for either pS-268 or p-tyrosine p120-catenin antibodies (green) shown independently in (E, G, I and K), or in a merge with E-cadherin antibody staining (red) and DNA/DAPI stain (blue) (F, H, J and L). (E, F, I and J) At E14.5, both forms of p120-catenin are expressed, with pS-268 strongly expressed in the periderm at the midline seam co-localizing with E-cadherin (E and F), while p-tyrosine clearly enriched in the area marking the border between the epithelial and mesenchymal populations (I, J, pink arrowheads). (G, H, K and L) At E14.75, pS-268 p120-catenin is strongly expressed in the epithelial islands and the oral and nasal epithelial triangles; this is co-localised with E-cadherin during EMT and endocytosis, while p120-catenin expression remains in some areas (H, white arrowheads). In contrast, p-tyrosine p120-catenin expression surrounds E-cadherin positive epithelial islands, while E-cadherin expression has disappeared in the intervening mesenchymal cells (L, pink arrowheads). Scale bars = 50 μm. Abbreviations: T, tongue; PS, palatal shelf.

Because all of our participants had either cleft palate or associated palatal anomalies, we also assessed p120-catenin expression during palatal fusion in the mouse, which occurs from embryonic day 12.5 (E12.5) to E15.5 (Fig. 5A–D). To examine this, we used two antibodies recognizing phosphorylated forms of p120-catenin: a tyrosine-phosphorylated form or phosphorylation at serine 268 (pS-268), which is proposed to trigger the disruption of epithelial cadherin-catenin complexes (52,53). Neither of these forms of p120-catenin had been previously analyzed in the palate. In palatal cross-sections at E14.5, the medial epithelial seam (MES) is evident (Fig. 5B), followed a few hours later with dissolution of the seam at E14.75 (Fig. 5C). While E-cadherin is expressed as expected in the MES (54) (Fig. 5F and J), the two forms of p120-catenin show very distinctive distributions. As the seam undergoes EMT, at E14.5, pS-268 is strongly expressed as predicted in cell–cell interfaces of the periderm layer along the medial seam, clearly co-localising with E-cadherin (Fig. 5E and F). As the seam degrades, E-cadherin expression is lost while p120-catenin expression remains (Fig. 5G and H, white arrowheads). To our surprise, we find phospho-tyrosine p120 staining in both the mesenchymal and the epithelial cells, with a clear enrichment marking the border between the epithelial and mesenchymal populations (Fig. 5I and J, pink arrowheads). This distribution appears unique to this stage of palate formation consistent with reports that p120-catenin is tyrosine phosphorylate in an EGFR-dependent manner (55), and continues during the degradation of the seam while E-cadherin expression decreases (Fig. 5K and L, pink arrowheads). As a control, in earlier stages (E11–12.5), the phospho-tyrosine expression is much lower and nearly identical to the pS-268 staining (data not shown).

### Heterozygous loss of p120-catenin leads to structural changes in the laryngeal apparatus

**Figure 6:**
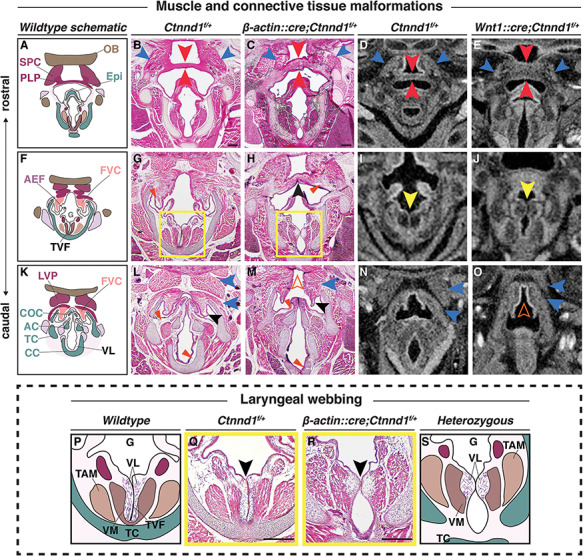
Heterozygous loss of p120-catenin leads to structural changes in the laryngeal apparatus. (**A**–**O**) Progression of the pharyngeal and laryngeal anomalies, (A, F and K) Schematics show the organization of the wild-type oropharynx from the more rostral (A) to caudal (K) planes. H&E staining of coronal sections through control (B, G, L: *Ctnnd1^fl/+^*) and heterozygous mutants (C, H, M: *β-actin::cre/+; Ctnnd1^fl/+^*) littermate at postnatal stage (P1). (B and C) The SPC (blue arrowhead) and PLP (red arrowhead) in mutants are disorganized with an increased thickness in the PLP cranio-caudally (C) as compared with the controls (B). (G and H) The FVC (vestibular folds) are well defined in the controls with abundant ligaments (G, red arrowhead). The FVC are fused in the mutant mice (H, black arrowhead) with ill-defined vestibular ligaments (H, red arrowhead). (L and M) The muscle attachments (blue arrowheads) superior to the FVC (black arrowhead) are well organized bilaterally in the controls surrounding the COC (L). Caudally, when the FVC separated in the mutants, it appeared hypoplastic (black arrowhead) as did the COC. The muscles (blue arrowheads) were ectopically fused to the LVP, producing an appearance of a ‘high-arched’ epiglottal area (M, orange hollow arrowhead). (D, E, I, J, N and O) Neural crest-specific mutants showed comparable laryngeal phenotype. μCT soft tissue scans of E16.5 control (D, I, N: *Ctnnd1^fl/+^*) or neural-crest-specific (E, J, O: *Wnt1::cre/+; Ctnnd1^fl/+^*) heterozygous mutant littermates. (D and E) Compare the PLP in control (D) to the very thick PLP muscle seen in mutant (E, red arrowheads). Compare the SPC in control (D) to the disorganized and hypoplastic SPC muscles seen in mutants (E, blue arrowheads). (I and J) Laryngeal webbing was observed in mutant TVF (J, yellow arrowhead) compared with parallel TVF in control littermate (I, yellow arrowhead). (N and O) Note aberrant muscle attachments (blue arrowheads) in (O) compared with control (N). Control (N) epiglottal region compared with the high-arched epiglottal area observed in mutant littermate (O, orange hollow arrowhead). (P–S) The laryngeal webbing phenotype. (P and S) Schematic representations of the wild-type (P) and mutant (S) anatomy at the vocal folds (TVF) from yellow-boxed insets in (G) and (H), respectively. (Q and R) H&E staining of coronal sections through control (Q: *Ctnnd1^fl/+^*) and heterozygous mutant (R: *β-actin::cre/+;Ctnnd1^fl/+^*) littermate at P1. (Q) In controls, well-defined VLs run parallel to the true vocal fold/cords (TVF). Underlying, the vocalis muscle (VM) and the thyroarytenoid muscle (TAM) are clearly attached and well-organised. (R) Laryngeal webbing is seen in the heterozygous mutant mice, where the VLs accumulate at a thin contact point (black arrowhead), thus perturbing the correct muscle attachments of the VM and TAM. Scale bars = 100 μm. Abbreviations: PLP, palatopharyngeus muscle; TAM, thyroarytenoid muscle; VM, vocalis muscle; HB, hyoid bone; Epi, epiglottis; OB, occipital bone; LVP, levator veli palatini muscle; AEF, aryepiglottic fold; FVC, false vocal cord; CC, cricoid cartilage; TC, thyroid cartilage; AC, arytenoid cartilage.

Some of our participants presented with anomalies associated with dysfunction of their velopharyngeal muscles and voice irregularities (Supplementary Material, Table S1; Table 2), a phenotype described in patients with VCF syndrome (56–58). Antibody staining confirmed the presence of p120-catenin protein during the development of the laryngeal and pharyngeal tissues in the mouse (Supplementary Material, Figure S3A). We then examined the laryngeal structures of mutant mice compared with their littermate controls at E16.5, P1 and P2.5 (Fig. 6). To do this, we crossed a mouse carrying the ubiquitous *β-actin::cre* driver with *Ctnnd1^fl/fl^* mice in order to generate heterozygous mutants (59,60) (Fig. 6C, H, M, R). Because we previously showed that the vocal ligaments (VLs) originated from the neural crest (61), we also generated tissue-specific *Ctnnd1* heterozygotes using the neural crest-specific driver, *Wnt1::cre* (62) (Fig. 6E, J, O). We found identical laryngeal anomalies in the heterozygous mutants in both mouse crosses, confirming the neural crest-specificity of these phenotypes.

Specifically, in control *Ctnnd1^fl/+^* mice, the palatopharyngeus (PLP) muscle, which elevates the larynx, is well defined and runs uniformly perpendicular to the epiglottis thereby attaching to the superior pharyngeal constrictor (SPC) muscle on either side (Fig. 6A, B and D). On the other hand, the PLP and the SPC were both severely disorganized in both sets of heterozygous mice with an apparent increase in the cranio-caudal thickness of the PLP muscle (Fig. 6C and E). Second, a striking phenotype known as laryngeal webbing was observed (compare controls, Fig. 6G, I, and Q, to mutants Fig. 6H, J, and R). Typically, the bilateral vocal cords are parallel and meet at the midline (Fig. 6F–G, with inset schematized and shown in Fig. 6P and Q). The outer layer of the vocal fold is made of an epithelium that encapsulates the lamina propria comprising the VLs (Fig. 6P and Q). These two layers function as the vibratory components for phonation and oscillation. Instead, in heterozygous mutant mice, the VLs show only a brief contact point between the opposing epithelia (Fig. 6H, with inset schematized and shown in 6R and 6S). The vocal cords are also thinner, lacking the lamina propria (Fig. 6R). Laryngeal webbing was also seen in the *Wnt1::cre* heterozygotes (Fig. 6J) compared with their littermate controls (Fig. 6I).

While the vestibular folds were well demarcated and the ligaments within them clearly defined in controls (Fig. 6G), the vestibular folds in the heterozygous mice were ectopically fused and the ligaments sparse and dispersed (Fig. 6H). Caudally, where the vestibular folds surrounded the normal corniculate cartilage (COC) (Fig. 6K and L), the folds have separated in the *Ctnnd1* heterozygotes, albeit hypoplastic (Fig. 6M). Similarly, the COC appeared hypoplastic and devoid of the underlying lamina propria (Fig. 6M). Finally, in mutants, the muscles were ectopically fused to the levator veli palatini muscles, which were then fused to the cranial base (Fig. 6M). This, in turn, gave the impression of a high-arched epiglottal area; a defect also found in the *Wnt1::cre* heterozygous mutants (Fig. 6O).

We also explored other craniofacial phenotypes in our heterozygous mouse model. Compared with their littermate controls (Supplementary Material, Figure S3B, a–e), mutant mice did not show any cleft lip (Supplementary Material, Figure S3B, f), face or limb dysmorphologies (Supplementary Material, Figure S3B, f–h) or cleft palate (Supplementary Material, Figure S3B, i) (*n* = 12), consistent with previous findings by Cox *et al.* This was confirmed by micro-computed tomography (μCT) to check for associated bony defects (*n* = 6) (Supplementary Material, Figure S3B, j).

### P120-catenin isoform 1 function is required in multiple organ systems

While the genetic mutation of *p120-catenin* in mouse models revealed a role for the neural crest in oropharyngeal development, analysis of multi-system involvement of p120-catenin was difficult due to the embryonic lethality of the homozygous null mice (5,9). We therefore turned to the frog *Xenopus*, where *in vivo* function of p120-catenin has been well studied (11,12,63). Previous analyses of p120-catenin requirements were mainly performed with antisense morpholino oligonucleotide knockdowns, which transiently prevent protein translation (11). Instead, to create genetic mutants, we used CRISPR*/*Cas9 approaches, allowing us to specifically delete different p120-catenin isoforms (64). As noted in the introduction, isoform 1 [full length at 968 amino acids (aa)] is most abundant in mesenchymal cells, while isoform 3 (start at aa 102) is preferentially expressed in epithelial cells (27–30). Isoforms 2 and 4, which start at 55 aa and 324 aa, respectively, are less well characterized.

**Figure 7:**
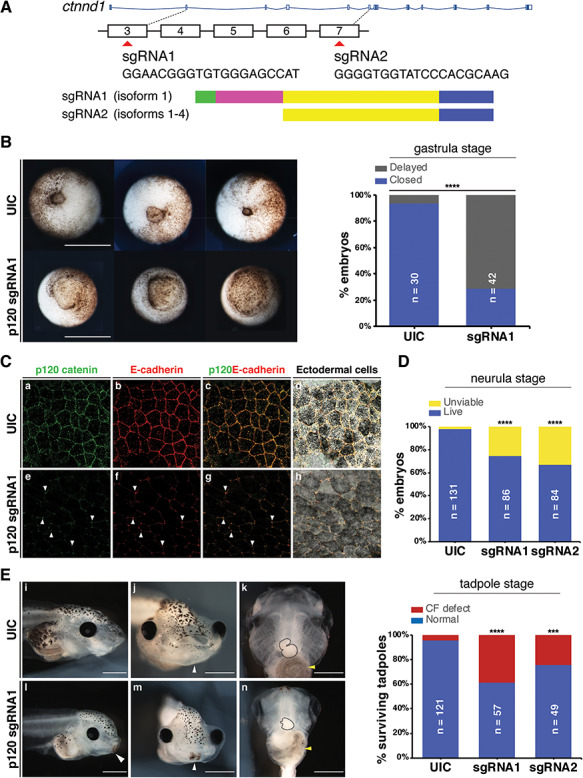
*Ctnnd1* knockouts in *Xenopus* give rise to craniofacial and heart defects. (**A**) Embryos were injected at the one-cell stage with sgRNAs, sgRNA1 and sgRNA2 targeting exons 3 and 7, respectively. (**B**) Ventral view showing blastopores at stage 11. Embryos injected with sgRNA1 had delayed blastopore closure (bottom row) compared to un-injected controls (UIC) (top row). The bar chart shows quantitation. Scale bars = 100 μm. (**C**) Confocal sections through the apical surface of ectodermal cells at stage 11 of embryos injected with sgRNA1 (e–h) and UICs (a–d). (C) (a–d) p120-catenin (a, green) is expressed in puncta at the cell membranes. E-cadherin (b, red) is expressed more evenly through the cell membranes. Both are colocalized at the cell–cell interface (c, d). Endogenous levels of p120-catenin and E-cadherin are diminished at the cell–cell interface in the sgRNA1-injected embryos (e, f). Residual p120-catenin and E-cadherin are seen in a spot-like pattern, only at the tricellular junctions (e–h, white arrowheads). (**D**) p120-catenin depletion led to lethality in embryos by the neurula stage. (**E**) Stage 46 tadpoles. (E) (i, l) Lateral views show a flattened profile in *p120* CRISPR tadpoles (l) compared with UICs (i). (E) (j, m) Frontal views showing a reduction in the size of mouth opening and a persistent cement gland (white arrowhead) in *p120* CRISPR tadpoles (m) compared with UICs (j). (E) (k, n) Ventral views showing a reduction in the size of craniofacial cartilages, altered cardiac looping (black-dashed outline) and altered gut coiling (yellow arrowhead) in *p120* CRISPR tadpoles (n) compared to UICs (k). Quantification of craniofacial defects in UIC and p120 depleted tadpoles. Scale bars = 100 μm. ^****^P < 0.0001; ^***^P < 0.001.

Embryos were injected at the one-cell stage with sgRNAs targeting either of two coding exons, exon 3 or exon 7 (sgRNA1 and sgRNA2 respectively, Fig. 7A). Disruptions in exon 3 are predicted to only affect isoform 1, while sgRNA2 targeting exon 7 disrupts all four isoforms.

When embryos were scored at gastrula stages following sgRNA1 injections, disrupted or delayed blastopore closure was evident (*n* = 30/42 versus 2/30 in the controls) (Fig. 7B). Furthermore, we noted severe early lethality (Fig. 7D), especially using sgRNA2 which blocked all isoforms (Fig. 7D). Notably, by neurula stages, the majority of these mutants died due to a loss of integrity in the epithelium (data not shown).

Since the most well-established epithelial role for p120-catenin is in complex with E-cadherin at cell–cell junctions, we first examined E-cadherin localization in the neurectoderm at stage 11, as gastrulation was concluding. Indeed, in uninjected controls, high levels of p120-catenin and E-cadherin were found co-localized at the cell interface (Fig. 7C, a–d). E-cadherin is expressed throughout the cell membrane (Fig. 7C, b), whereas p120-catenin, though localized to the cell membrane, appears distributed in puncta (Fig. 7C, a). Upon p120-catenin deletion, the expression levels of endogenous E-cadherin in the epithelial cells were diminished particularly at the interface between the cells, leaving only the spot-like localization of both proteins at the tricellular junctions of these epithelial cells (Fig. 7C, e–h). The residual expression of p120-catenin may be due to the maternal loading of the protein, as the CRISPRs should only affect zygotic transcription, or due to the mosaicism of the CRISPR deletion.

**Figure 8:**
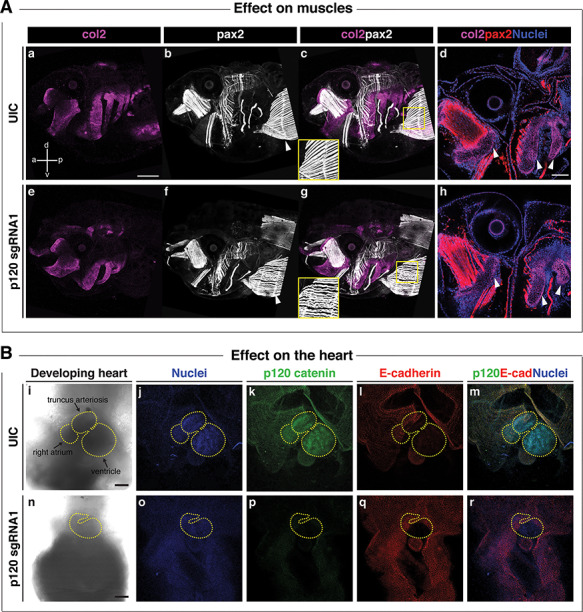
*Ctnnd1* knockouts in *Xenopus* give rise to altered morphogenesis of the muscles and heart. (**A**) Immunofluorescent staining for collagen 2 (col2, magenta), muscle/pax2 (white) and nuclei (DAPI, blue); (a, anterior; p, posterior; d, dorsal; v, ventral). (A) (a, e) A lateral view of col2-positive branchial cartilages in UIC (a) and *p120* CRISPR mutant (e) reveals the hypoplasia of mutant cartilages; however, cell morphology appears normal in *p120* CRISPR mutants (h) (d and h, white arrowheads). (A) (b, c, f and g) Pax2-expressing muscles revealed a defect in the fibril organization of the rectus abdominus muscle in the *p120* CRISPR tadpoles (f, white arrowhead) compared with the UIC muscles (b, white arrowhead); note insets in (c, g). (**B**) Ventral views of hearts of stage 46 tadpoles. Immunofluorescent staining for p120-catenin (green), E-cadherin (red) and DNA (blue). (B) (i–m) Controls; (n–r) *p120* CRISPR mutant tadpoles. Morphologic defects are evident in the size of the heart and directionality of the loops (compare control heart (i) to mutant heart (n), yellow-dashed outlines). (B) (k, p) p120-catenin is strongly expressed in the heart of UIC tadpoles (k) but is lost in *p120* CRISPR tadpoles (p). (B) (l, q) Note the absence of E-cadherin in the control and mutant hearts. Scale bars = 100 μm.

As the sgRNA2 CRISPR was predicted to disrupt all four isoforms and led to severe lethality by neurula stages, the majority of analyses were performed using the sgRNA1 CRISPR, which is predicted to disrupt the predominantly mesenchymal isoform 1. A proportion of the knockout animals survived past the neurula stages, possibly due to mosaicism, and were examined at stage 46 to determine whether craniofacial and organ development had occurred normally. We observed obvious craniofacial defects in the CRISPR mutants, including a reduction in the width and height of the head (Fig. 7E, l–n), a hypoplastic mouth opening (Fig. 7E, m), delayed breakdown of the cement gland (Fig. 7E, l, m) and heart and gut looping anomalies (Fig. 7E, n). Following on from the disorganization of the laryngeal muscles seen in the mouse mutants (Fig. 6), antibody staining against Pax2 was used to label the muscle fibers while anti-collagen 2 (col2) antibody labelled craniofacial cartilages in the mutants (Fig. 8A, a–h). In control animals, the muscle fibers were well organised and straight, while in the mutants, the muscle morphology appeared disorganized, particularly the rectus abdominus muscle, with muscle striations being replaced by irregularly shaped fibers (Fig. 8A, f, g). Consistent with previous observations (Fig. 7), craniofacial cartilages were hypomorphic and compacted both in the anterior-posterior and dorsal-ventral axes (Fig. 8A, a and e). However, morphology of the chondrocytes appeared normal (Fig. 8A, d, h).

Finally, since the participants (6/13) had a high frequency of congenital heart defects and because p120 is strongly expressed in the heart of human, mouse and frog embryos, we examined the hearts in the CRISPR-knockout tadpoles. Notably, the strong expression of p120 seen in the different heart chambers in the control tadpoles was lost when p120 was knocked down (Fig. 8B, p). The majority of mutant tadpoles had heart anomalies including heart-looping defects (Figs 7E, n and 8B, n). Notably, E-cadherin is not expressed in the normal heart or the muscles (Fig. 8B, I), suggesting that the heart and muscle phenotypes may be manifestations of E-cadherin independent functions of p120.

## Discussion

This work expands upon the spectrum of abnormalities associated with *CTNND1* variants beyond non-syndromic CLP and BCD (1–3). Most notably, we describe in detail characteristic craniofacial features including choanal atresia and unusual patterns of hypodontia as well as heart, limb, laryngeal and neurodevelopmental anomalies. We find the expression of *CTNND1* mRNA during the development of the pharyngeal arches in human embryos, and we define the profile of two phosphorylated forms of p120 in the mouse palate. Finally, genetic approaches in mouse and *Xenopus* demonstrated novel roles for *CTNND1* in the oropharynx, craniofacial cartilages and in the heart. Thus, our data implicate *CTNND1* variants as causative of a broad-spectrum syndrome that overlaps with DiGeorge VCF syndrome as well as other disorders of craniofacial development such as CHARGE and Burn McKeown syndromes (65–68). All of these syndromes could be collectively considered to be neurocristopathies. Notably, the neural crest-specific disruption of *CTNND1* in our animal models supports this role for *CTNND1* as a candidate neurocristopathy gene, and we suggest that these newly identified variants likely highlight both epithelial and mesenchymal roles for p120-catenin.

Prior to our study, the majority of the participants did not have a recognizable or a diagnosed condition when they were recruited. Here, we demonstrate that they collectively share consistent characteristic phenotypic features that suggest that mutations in *CTNND1* may lead to a much broader phenotypic spectrum than previously described (1,2). For instance, low set ears were reported in one case of BCD by Kievit and colleagues (1); we find multiple participants with auricular anomalies particularly the low-set ears and over-folded helices (Supplementary Material, Figure S1B; Supplementary Material, Table S1). Similarly, syndactyly was reported in one of the *CTNND1* patients described in Ghoumid *et al*. (2), and clinodactyly (one patient) and camptodactyly (two patients) were reported by Kievit *et al*. (1). Again, we find limb anomalies consistently associated with *CTNND1* variation (Supplementary Material, Figure S1C; Supplementary Material, Table S1). The cardinal features of BCD include the ectropion of the lower eyelids, euryblepharon and lagopthalmos (69,70); these were not evident. However, five of our patients showed other BCD-eyelid manifestations such as distichiasis and ankyloblepharon (Supplementary Material, Table S1); we also found short up-slanting palpebral fissures, hooded eyelids, high arched eyebrows and telecanthus (Supplementary Material, Figure S1A; Table 2 and Supplementary Material, Table S1). As BCD is associated with both *CTNND1* and *CDH1* (E-cadherin) variants, some of these phenotypes may represent the distinctive functions of the E-cadherin-p120 complex; the majority of these functions could be attributed to a role for the cadherin-catenin in epithelia (71).

Of note, eight individuals had severe hypodontia, including missing permanent canines and first permanent molars, even in those without cleft lip/palate. Thus, missing canines and molars could be classified as a microform cleft anomaly, especially when found in association with high-arched palate (72) (Fig. 3I and K; Supplementary Material, Table S2).

Beyond the known phenotypes associated with *CTNND1* and *CDH1*, we note the novel phenotypes seen in our patients, which include the heart anomalies and behavioral disorders. These have not been reported previously in patients with a BCD diagnosis. Nevertheless, our findings suggest that both *CTNND1* and *CDH1* should be tested in patients with congenital orofacial and cardiac anomalies. A key finding was choanal atresia in four individuals; given the rarity of this anomaly, both *CTNND1* and *CDH1* should be considered during genetic profiling of patients with this anomaly, in addition to CHARGE and other syndromes noted above. Indeed, Nishi *et al*. (73) reported cleft lip, right choanal atresia, a congenital cardiac anomaly (tetralogy of Fallot), agenesis of the corpus callosum, up-slanted palpebral fissures and ear anomalies in a patient with *CDH1* mutation; however, at the time, this was not diagnosed as BCD.

While all of the variants found in the present study resulted in the truncations of p120-catenin, they fell broadly into three distinct groups: those falling within the N-terminal regulatory region (p.Val148Aspfs^*^24), those disrupting the armadillo repeat region and presumably subsequent interactions with E-cadherin (e.g. p.Arg461^*^, p.Arg797^*^, p.Leu494Argfs^*^5 and p.GLy532Alafs^*^6) and those falling in the C-terminal domain (p.Ser868^*^, the splice variant c.2702-5A>G and p.His913Profs^*^3). Those falling in the N-terminal region would be predicted to have the most complete deletion and to best mimic a heterozygous loss of function situation. Indeed, similar to the heterozygous mice, these subjects did not have cleft palate. Interestingly, those probands with C-terminal truncations had the most complete cleft lip and palate phenotypes. This was consistent with previous reports by Kievit *et al*. (1), who reported a non-sense mutation (p.Trp830^*^), and Cox *et al*. (3), who reported p.Arg852^*^ and a splice site mutation (c.2417+G>T) (3). As these C-terminal truncations would all be predicted to retain E-cadherin binding but lose crucial RhoGAP interactions (24), one might hypothesize that a mutation in this region prevents p120 clearing from the epithelial complex, which is necessary for seam dissolution during palate closure. Therefore, future analyses should focus on whether these C-terminal truncations are acting in a dominant-negative manner, and preventing clearance of E-cadherin from the seam. It would also be useful to check whether any variants lead to non–sense-mediated decay, especially those lying in the armadillo domain, as Kievit and colleagues demonstrated (2).

**Figure 9:**
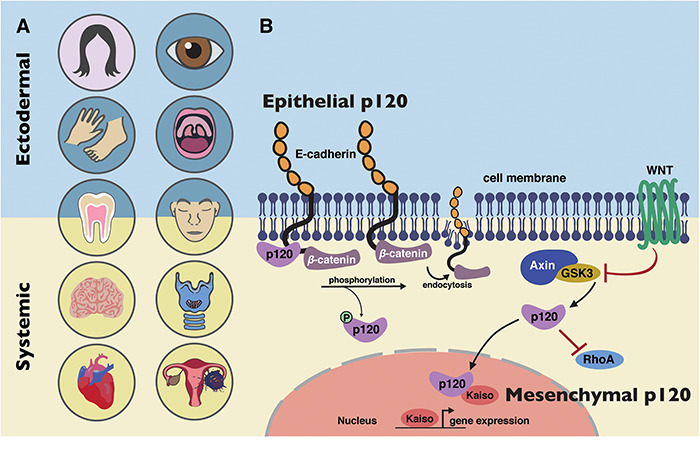
Model of *CTNND1* function in systemic disease. (**A**) *CTNND1* mutations are not only implicated in conditions that affect epithelial structures but also systemic conditions that originate from mesenchymal roles of p120-catenin. Structures in pink circles have been described in previous publications on *CTNND1* (1,2); structures in blue circles have been implicated previously in *CTNND1*-related disorders (1,2) and in this study. Structures in yellow circles have been identified in this study. (**B**) BCD is primarily due to disturbances in E-cadherin/p120 interactions. The inclusion of other organ systems described here highlights the involvement of other known molecular functions of p120, such as its role in the WNT signalling pathway and its interactions with Rho-GTPases, demonstrating its mesenchymal roles in producing these systemic conditions.

With regards to non-epithelial functions of p120, some of the phenotypes that this study, and others, has reported could be explained by the known interactions of p120 in the Wnt signalling pathway (20). Epithelial-specific knockouts of p120 (using a *keratin-14* promoter) did not show tooth agenesis (10), suggesting that the tooth anomalies in our patients do not arise from the epithelial functions of p120. In support of this, two key genes implicated in tooth agenesis are the Wnt ligand, *Wnt10A* and a Wnt target gene *Axin2* (74–84). The Wnt signalling pathway may also explain the laryngeal findings (Fig. 6), as the knockout of the Wnt transducer β-catenin is also known to lead to similar vocal fold anomalies (85) as those seen in our neural crest-specific *p120-catenin* heterozygotes (Fig. 6). Furthermore, by targeting isoform 1, we should be losing the mesenchymal form of p120 (Figs 7 and 8). These findings are consistent with prior studies focused on neural crest, where the p120-catenin association with Wnt signalling is well established (32,86,87). Thus, we hypothesize that a subset of p120 phenotypes can also be attributed to Wnt perturbation in the neural crest (Fig. 9).

The heart defects seen in our patients could also be attributed to a failure in neural crest development, which is known to be crucial for the development of the septum and valves (88–92). Congenital heart disease (CHD) is the most common human birth defect with an incidence that varies between 0.8 and 2% in neonates (93,94). In addition, mild anomalies of the heart may be undiagnosed. In contrast, we noted that nearly half the subjects in our cohort have some form of CHD (6/13). Of the CHD incidences seen in live births, phenotypes can range from minor septal defects to severe malformations requiring lifesaving surgical repair. A similar range of anomalies was reflected across our cohort (Table 2). Furthermore, within families with single gene mutations, different types of structural cardiac malformations may be observed (93,94). For example, we observed this in Family A, where both mother and daughter have septal defects but additional presentations vary (one has a hypoplastic aortic arch and mitral valve stenosis while the other has patent ductus arteriosus, Table 2). Thus, a thorough survey of phenotypic variation and the inclusion of mild anomalies is important for generating new hypotheses on key roles for CTNND1 during human development. Future analysis will be necessary to definitely determine functional relevance of the different isoforms and the relationship with our novel patient variants.

In addition to the phenotypes shared commonly across our cohort, some participants in this study had scoliosis, and one family reported two deceased children, who had bifid uvula, congenital cardiac disease (VSD, PDS), eye anomalies, developmental delay and chronic bowel immotility and gastroesophageal reflux disease; however, no genetic testing had been carried out. One patient presented at a young age with an ovarian dysgerminoma. To our knowledge, this is the first patient with a *CTNND1* variant associated with an early onset cancer. Though p120 and E-cadherin have been associated with cancer and tumourigenesis (23–25,93–95), our data are insufficient to determine linkage in this case. Nevertheless, future studies of genetic cancers should consider E-cadherin/p120-catenin status as well.

Finally, a number of patients reported in DECIPHER have copy number variants (CNVs) affecting *CTNND1* (data not shown). Previously published missense mutations are noted in Figure 1. Interestingly, for CNVs, both deletions and duplications have been associated with partially overlapping phenotypes. For instance, two patients with a deletion of less than 4 MB had anomalies including bulbous nose, limb anomalies, delayed speech and language development, intellectual disability, nasal speech, ventricular septal defect and cleft lip (data not shown). Further studies will be necessary to understand the functional consequences of these genetic changes.

In summary, we demonstrate that for the first time, p120 is not only involved in human conditions involving epithelial integrity, most likely caused by aberrant E-cadherin/p120 interactions, but also in other important intracellular functions (Fig. 9). We conclude that *CTNND1-*related disorders span a spectrum of phenotypes ranging from multi-system involvement to non-syndromic clefting. While further studies will be necessary to definitively understand the phenotype-genotype correlations, *CTNND1*, and perhaps *CDH1*, should be considered when patients present with characteristic craniofacial anomalies, congenital cardiac defects and neurodevelopmental disorders.

## Declarations

S.A.L. is part owner of Qiyas Higher Health, a startup company unrelated to this work.

## Supplementary Material

Alharatani_p120_SupplFigLegends_ddaa050

AlharataniSupplTable1_ddaa050

AlharataniSupplTable2_ddaa050
