# Supplementary material for: Novel truncating mutations in CTNND1 cause a dominant craniofacial and cardiac syndrome
Source: Hum Mol Genet. 2020 Mar 20;29(11):1900–21. doi: 10.1093/hmg/ddaa050 (PMC7372553; doi:10.1093/hmg/ddaa050)
Supplement: Alharatani_p120_SupplFigLegends_ddaa050 [file alharatani_p120_supplfiglegends_ddaa050.doc]

***Supplemental Figure 1. Clinical presentation of individuals with a CTNND1 mutation****.*

[A] The eye phenotypes of the narrow palpebral fissures, the hooded eyelids and highly arched, thin lateral eyebrows were evident from a young age. [B] Ear anomalies included: low-set ears, sometimes asymmetric and/or small; overfolded helices of the external ears; a pre-auricular pit was also seen in one of the patients (data not shown). [C] Upper limb anomalies included: slightly shorter 5^th^ fingers as seen in Patients 3, 12 and 13; and a single transverse palmar crease on the right hand seen in both Patients 3 and 8. Lower limb anomalies included: 2,3-cutaneous syndactyly of the feet; sandal gaps and camptodactyly of the 2^nd^ toe as seen in Patients 12 and 13; a longer 4^th^ toe in Patient 6 and short toes in Patient 7.

***Supplemental Figure 2. CTNND1 is expressed during relevant stages of human embryonic development.***

[A] Coronal cross-section through the torso at CS21. [B] *CTNND1* is expressed in the columnar epithelial lining of the stomach wall and continues through the pyloric part of the stomach. [C] Expression is seen in the islet of Langerhans in the pancreas. [D] Expression in the germinal center of the spleen. [E-G] Progressing caudally through the pelvis, *CTNND1* is expressed in the epithelial lining of the bladder [E], the rectum/hindgut [F], the spinal cord and vertebral body (VB) [G].

***Supplemental Figure 3. Mouse p120-catenin is expressed in the epithelial and mesenchymal compartments of the laryngeal and pharyngeal apparatuses.***

[A] Immunohistochemistry using the anti-phosphotyrosine p120-catenin antibody on sagittal sections through wild-type mice at E13.5 (a-a’) or E15.5 (b-b’). [a, b] Positive staining is seen in the epiglottis, esophagus and the larynx. [A] (a’, b’) Insets from (a and b, respectively). Muscles that express p120-catenin in the laryngeal and pharyngeal apparatuses are shown (pink arrowheads). Abbreviations: E, epiglottis; Oe, entrance to oesophagus; L, laryngeal auditus; H, heart; SP, soft palate; TC, thyroid cartilage; CC, cricoid cartilage; T, tongue; Tr, trachea

***Heterozygosity in p120-catenin leads to normal facial and oral phenotypes.***

[B] Shown are postnatal P2.5 mice. Heterozygous mutant *β-actin::cre/+;Ctnnd1^fl/+^* mice do not exhibit facial or lip anomalies (f-g) and are comparable to littermate controls (a-b). [B] (c, h) No limb anomalies are observed. [B] (d, i) Postnatal P1 mice. Intra-oral views of the palate of wild-type (d) and heterozygous mutant littermate (i), cleft palate defects were not observed. [B] (e, j) Microcomputed tomography (µCT) scans showed normal palates in P2.5 control (e) and heterozygous mutant littermate (j).

**Supplementary Table S1:** Detailed phenotyping of Individuals with a *CTNND1* variant.

**Supplementary Table S2:** Reported congenitally missing teeth.
