# Supplementary material for: Novel truncating mutations in CTNND1 cause a dominant craniofacial and cardiac syndrome
Source: Hum Mol Genet. 2020 Mar 20;29(11):1900–21. doi: 10.1093/hmg/ddaa050 (PMC7372553; doi:10.1093/hmg/ddaa050)
Supplement: AlharataniSupplTable1_ddaa050 [file alharatanisuppltable1_ddaa050.pdf]

Table S1. Clinical details of individuals with a CTNND1 variant

Participant	Patient 1	Patient 2	Patient 3	Patient 4	Patient 5	Patient 6	Patient 7	Patient 8	Patient 9	Patient 10	Patient 11	Patient 12	Patient 13	TOTAL	Kievit et al 2018	Ghoumid et al 2017
**DDD ID**	**NA**	**NA**	**294023**	**278766**	**NA**	**-**	**278000**	**264418**	**NA**	**NA**	**265667**	**281120**	**-**	**-**	**-**	**-**
**Variant**	V148Dfs*24	V148Dfs*24	R461*	R461*	R797*	L494Rfs*	L494Rfs*	G531Afs*6	S868*	S868*	c.2702-5A>G	H913Pfs*3	H913Pfs*3	_	_	_
**Sex**	Female	Female	Female	Male	Female	Female	Male	Female	Male	Male	Male	Male	Male	6F/7M	_	_
**Craniofacial**																
Cleft lip/palate	no	no	submucous cleft soft palate & bifid uvula	no	no	yes	right unilateral cleft lip and palate	no	bilateral cleft lip and palate	right unilateral cleft lip and palate	left unilateral cleft lip and palate	left unilateral cleft lip and palate	left unilateral cleft lip and palate	**(8/13)**	Yes	Yes
High arched palate	yes	yes	yes	no	no	no	yes	yes	no	ND	_	yes	yes	**(7/13)**	NR	NR
Thin upper lip	yes	yes	no	no	no	no	yes	yes	yes	no	yes	yes	no	**(7/13)**	NR	NR
Choanal atresia	bilateral	left	no	no	no	no	no	bilateral	no	no	right	no	no	**(4/13)**	NR	NR
Dysplastic ears	_	low set ears	asymmetric low set ears; overfolded helices.	slightly anteverted	no	small	small	low set ears; overfolded helices.	low set; overfolded helices.	low set; overfolded helices.	preauricular right pit, mild conductive hearing loss bilaterally.	_	_	**(9/13)**	Yes, 1 subject	NR
Wide nasal bridge	yes	yes	yes	no	no	yes	yes	yes	yes	yes	yes	yes	yes	**(11/13)**	NR	NR
Broad nasal tip	yes	no	yes	no	no	no	yes	yes	yes	yes	yes	no	no	**(7/13)**	NR	NR
Mid-face hypoplasia	yes	yes	yes	no	no	yes	yes	yes	no	no	yes	yes	yes	**(9/13)**	No	NR
Mandibular prognathism	yes	no	yes	no	no	no	no	yes	no	no	yes	no	yes	**(5/13)**	No	NR
Brachycephaly	no	brachycephaly	no	plagiocephaly & brachycephaly	no	no	no	no	no	no	brachycephaly	no	no	**(3/13)**	NR	NR
**Eyes and eyelids**																
	eyelid synechiae bilaterally, distichiasis & mild ectropion.	ankyloblepharon bilaterally, distichiasis & hyperopia.	narrow upslanted palpebral fissures, hooded eyelids and telecanthus; no ophthalmic concerns.	narrow upslanted palpebral fissures, hooded eyelids and telecanthus, absent eyelashes medially, mild ectropion (everted upper and lower eyelids laterally).	wide palpebral fissues with scleral show, hypertelorism, ectropion, distichiasis.	sleeps with eyes open; ectropion-everted outer third of the lower eyelids.	narrow palpebral fissures, ankylopblepharon, sleeps witheyes open, watery eyes, distichiasis.	narrow upslanted palpebral fissures, hooded eyelids; had a squint. No ophthalmic concerns.	narrow palpebral fissures, hooded eyelids; bilateral eyelid tags at birth, ?ankyloblepharon, telecanthus.	narrow upslanted palpebral fissures, hooded eyelids;telecanthus.	narrow upslanted palpebral fissures, hooded eyelid and telecanthus. Nasolacrimal obstruction: left side complete, right side partial. No other ophthalmic concerns.	narrow upslanted palpebral fissures, hooded eyelids and telecanthus. No ophthamic concerns.	narrow upslanted palpebral fissures, hooded eyelids and telecanthus. No ophthalmic concerns.	**Narrow palpebral fissures (9/13); hooded eyelids (8/13); telecanthus (7/13); mild ectropion (4/13); distichiasis (4/13); ankyloblepharon (3/13)**	Reported BCD eyelid malformations	Reported BCD eyelid malformations
Highly arched eyebrows	yes	yes	no	no	no	yes & bushy eyebrows	yes & interrupted eyebrows	yes	no	no	yes	yes	yes	**(8/13)**	NR	NR
Thin lateral eyebrows	yes	no	no	no	yes	yes	yes	yes	yes	yes	yes	no	no	**(8/13)**	NR	NR
**Dental/oral**																
Hypodontia	yes, 9 permanent teeth	yes, 5 primary & permanent; other premolars could not be assessed at this age.	yes, 12 permanent teeth	yes (number not determined)	yes, 3 permanent teeth	-	-	yes, 8 permanent teeth	? missing back molars, ND	? missing back molars, ND	yes, 3 permanent teeth	no	yes, 3 permanent teeth	**(8/13)**	Yes	Yes
Delayed dentition	yes; supereruption of 63 with apical resorption from 24. Delayed development 34 & 44 with ankylosed submerged and resorbed 84 .	yes, impacted 63 and delayed eruption of the second primary molars	_	yes	yes	-	-	ND	ND	ND	yes, ectopic 23	no	delayed development of 21	**(6/13)**	Yes	NR
Abnormal crown form	yes; mesoangular eruption of 24, fusion of teeth, long cone-shaped tooth.	yes; macrodontia of 63 & 75; supernumerary tooth in 64-65 area; tooth 51 is dysmorphic and diminutive and tooth 73 is dysmorphic and rotated.	yes; peg 22	no	yes, multiple conical/peg teeth.	-	disorganised teeth	yes; peg 21, 22 and history of neonatal tooth.	'pointy teeth'	ND	yes; fissured incisors	yes; diminutive 22	no	**(9/13)**	Yes	Yes
**Limbs**																
Hands	NAD	NAD	single transverse palmar crease, right hand; thumbs and fifth finger slightly shorter.	NAD	NAD	small hands; short fifth finger.	small hands; short fifth finger.	single transverse palmar crease, right hand.	NAD	NAD	mild syndactyly and camptodactyly	slightly shorter fifth finger	slightly shorter fifth finger	**(7/13)**	No	No
Feet	NAD	slight 2,3-syndactyly of feet bilaterally, long halluces.	slight 2,3-syndactyly on feet, lateral deviated halluces.	pes planus, hallux valgus.	NAD	4th toe longer than others, schilles stretched as a child; 2,3-syndactyly on right foot.	short toes	NAD	NAD	NAD	NAD	sandal gap, camptodactyly 2nd toe.	sandal gap, camptodactyly 2nd toe.	**(7/13)**	Yes	Yes
**Systemic Phenotypes**					-											
**Cardiac**	hypoplastic aortic arch, ASd, VSd, MVS.	VSd, PDA, PFO.	NAD	VSd, secundum ASd.	NAD	NAD	NAD	TOFt - repair done at 4 months, pulmonary regurgitation - valve replacement at 9 years.	NAD	NAD	hypoplastic aortic arch with severe CoA; perimembranous VSd; surgeries at 1 month & 2 years.	VSd	NAD	**(6/13)**	No	No
**Neurologic**	NAD	sensory processing disorder. Query autism & ADHD, GDD; aggressive behaviour.	speech delay early life, autism & ADHD, LD, defiant behaviour.	autistic traits, poor coordination.	NAD	NAD	NAD	NAD	incresingly challenging behaviour; anxiety, parental concern re:autistic traits.	increasingly challenging behaviour; speech and language delay; developmental delay.	severe speech and language delay; autism; mild LD; OCD.	mild LD.	mild LD.	**(8/13)**	No	No
**Voice anomaly**	no	nasal speech	no	no	ND	nasal speech	no	no	no	no	stridor; hoarseness	no	no	**(3/13)**	No	No
**Other skeletal anomalies**	severe scoliosis	-	delayed fontanelle closure	joint laxity	-	scoliosis as an adult; short stature	-	-	-	-	short stature; asymetric shoulder; short neck	-	-	**(5/13)**	No	No
**Cancer**	no	no	no	no	no	no	no	ovarian dysgerminoma at 12 years of age stage III in the left ovary; underwent left oophorectomy followed by chemotherapy.	no	no	no	no	no	**(1/13)**	No	No
**Others**	restrictive lung disease	partial agenesis of the corpus callosum	velopharyngeal insufficiency; early onset puberty; bowel problems infant-toddler years	-	-	hypothyroid	abnormal nasal morphology: had rhinoplasty for a collapsed nose	macroglossia (enlarged tongue)	glue ear	glue ear	cryptorchidism on the left side; multiple grommets	coronal hypospadias (repaired); monozygotic twin of Patient 13	monozygotic twin of Patient 12	_	No	No

Abbreviation: DDD ID, Deciphering Developmental Disorders patient identification number; NA, not applicable; NAD, no abnormality detected, ND; not determined because of non-availability; 23, upper left permanent canine; 63, upper left primary canine; 24, upper left permanent first premolar; 34, lower left permanent first premolar; 44, lower right permanent first premolar; 84, lower righ primary first molar; 22, upper left permanent lateral incisor; 21, upper left permanent central incisor; 75, lower left primary second molar; 73, lower left primary canine; 64, upper left primary first molar; 65, upper left primary second molar; VSd,
