# Supplementary material for: Novel truncating mutations in CTNND1 cause a dominant craniofacial and cardiac syndrome
Source: Hum Mol Genet. 2020 Mar 20;29(11):1900–21. doi: 10.1093/hmg/ddaa050 (PMC7372553; doi:10.1093/hmg/ddaa050)
Supplement: AlharataniSupplTable2_ddaa050 [file alharatanisuppltable2_ddaa050.pdf]

Table S2. Reported congenitally missing teeth

Patient ID	Missing teeth
Patient 1	*16*, 15, **23**, 25, *26*, *36*, **35**, 45, *46*
Patient 2	54, 84 and **23**, *36*, 44
Patient 3	15, 14, 12, 11, 21, 24, 25, **35**, 31, 41, 44, 45
Patient 5	23,25, 45
Patient 8	*16*, 15, **23**, 25, *26*, *36*, **35**, 45
Patient 11	15, **35**, 45
Patient 13	22, **35**, 45

Missing permanent canines are in bold and missing permanent first molars are in italics.
